# Older LGBTQ Women Matter: An Examination of Distinctive Histories, End-of-Life Needs, and Support

**DOI:** 10.1093/geroni/igaa057.2066

**Published:** 2020-12-16

**Authors:** Korijna Valenti

## Abstract

LGBTQ (lesbian, gay, bisexual, transgender, and queer) aging research has reached a point at which in depth examination of the heterogenous experiences of identity, social life, and health is needed in order to better understand the distinct life courses and service needs of diverse subgroups. Older LGBTQ women have diverse life experiences and areas of need in terms of professional, social, and health-related supports and systems. As emerging literature addresses the depth of these distinctions, issues of identity development, social isolation, social networks, and end of life (EOL) planning and expectations require further study as well as the use of varied methods of data collection and analysis to enhance empirical understanding. This symposium includes findings from three studies. In a qualitative study, Valenti and colleagues examined experiences of LGB women over60 who had recently lost a spouse or partner and explored issues related to preparation and expectations at the EOL. In a discourse analysis of personal aging narratives published in the Bi Women’s Quarterly newsletter (including poetry, personal reflections, and short stories), Jen and colleagues examined how bisexuality is experienced and constructed through language use in context. Rowan and colleagues provide insights from interprofessional research with a specific focus on lesbian women in later life. All three presentations in this symposium illustrate how language, individual preferences and needs, and social support issues meet to inform the needs of LGBTQ older women as well as providing implications for future aging research, theory development, and interventions in practice.

